# Pediatric collaborative care outcomes in a regional model

**DOI:** 10.3389/fpsyt.2023.1252505

**Published:** 2023-11-22

**Authors:** John T. Parkhurst, Catherine Garcia-Goetting, Eric Peist, Rachel Ballard, Courtney Romba, John V. Lavigne

**Affiliations:** ^1^Ann & Robert H. Lurie Children's Hospital of Chicago, Chicago, IL, United States; ^2^Northwestern University Feinberg School of Medicine, Chicago, IL, United States

**Keywords:** primary care, collaborative care, mental health, measurement based care, pediatric, primary care integration and behavioral health

## Abstract

**Background:**

Despite the movement toward hospital-based medical centers acquiring pediatric primary care offices, many primary care pediatricians still work in small, independent practices. To expand mental healthcare access, service delivery models must consider primary care practice needs and regionally available resources.

**Objective:**

This report describes the implementation and evaluation of the Mood, Anxiety, ADHD Collaborative Care (MAACC) program over a 4 years period. MAACC. MAACC engaged 97 pediatric primary care clinicians across 39 practices in mental health training and supported the treatment of referred patients through a collaborative care model. To support psychosocial treatment needs, we built a child community therapy referral network of 213 licensed psychotherapy providers.

**Methods:**

Data were collected on service delivery patterns (e.g., referrals, treatment use, and attrition) and patient outcomes. Measures included parent and children and adolescents PROMIS anxiety and depression short forms and the Parent NICHQ Vanderbilt.

**Results:**

Six hundred ninety-six children and adolescents aged 6–18 were evaluated and provided treatment recommendations. Anxiety disorders were the most common diagnosis (45.4%), followed by ADHD (30.7%) and mood disorder (17%). For children and adolescents with an anxiety or mood disorder, significant improvement was observed from baseline to any initial follow-up and from baseline to 6, 12-, and 18 weeks on children and adolescents and parent measures of anxiety and depression. For children and adolescents with ADHD, significant improvement was observed from baseline to any initial follow-up measure and at 6 and 18 weeks on parent-reported inattentive symptoms. Significant differences in treatment outcomes were identified for children and adolescents with anxiety receiving psychotherapy alone and medication management and psychotherapy.

**Conclusion:**

MAACC utilization and patient outcomes suggest that real-world collaborative care can effectively provide high-quality care while cultivating increased primary care treatment capacity and building on existing community resources.

## 1 Introduction

The prevalence of children with at least one mental health condition is 16.5% (1), and the unmet need for mental health treatment is considerable (2). Because the United States has not sufficiently developed a systematic approach to the children and adolescents mental health crisis (3), there is a critical need to expand the capacity of the existing workforce and to develop systems of high-quality and accessible mental health treatment.

Pediatric primary care clinicians (PPCC) are a critical element of the mental health workforce (4, 5). To facilitate mental health treatment in primary care, additional training and collaborative or integrated care models can be employed (6, 7). The collaborative care model (CoCM) has strong evidence of improved time to treatment, better patient outcomes, and long-term healthcare savings in mental health treatment for adults (8–10). CoCM has also demonstrated the clearest clinical benefit for pediatric patients considering the effectiveness of research on other available primary care integration models (11–13). CoCM includes core components such as: (a) population-based care for a defined group of patients; (b) patient-centered collaboration; (c) use of evidence-based treatment approaches; (d) use of measurement-based “treatment to target”; and (e) accountable care based on setting elements. Yonek et al. identified that three of these components—population-based care, measurement-based care, and access to evidence-based mental health services—were most commonly associated with clinical improvement in Randomized Control Trials of collaborative models (14). The deployment and study of CoCM and other collaborative care models in real-world primary care settings are evolving (10, 15, 16). It is also recognized that collaborative models may often need to be modified or adjusted to unique primary care settings to reduce implementation barriers and leverage context-dependent resources (14, 17).

The Mood, Anxiety, ADHD Collaborative Care (MAACC) program was developed as a hub-spoke (18) approach by which a medical center could support the mental health needs of small, independently owned, primary care practices. In a needs assessment, pediatric practitioners indicated their greatest needs were to: (a) improve access to mental health treatment for children and adolescents aged 6–18 being treated in pediatric primary care; (b) increase timely, patient-centered access to psychotherapy; and (c) optimize evidence-based treatment by improving collaboration with mental health specialists. MAACC incorporated additional objectives of employing measurement-based care and tracking reimbursement. Results of the 15 months study of the effectiveness of MAACC identified significant patient- and parent-reported symptom improvement in ADHD, mood, and anxiety disorder symptoms. Confidence in treatment provision and perceptions of access to care substantially improved after PPCCs were engaged in MAACC (18).

MAACC adapted access to evidence-based psychotherapy care, often referring psychotherapeutic care to locally available psychotherapists to provide psychosocial treatment. Medication treatment and psychotherapeutic treatment for children and adolescents commonly occur in outpatient settings (4, 19). In the current mental health system, outpatient psychotherapeutic care typically consists of private practices unlinked to primary care practices. While community psychotherapy practices may vary in the populations served and the use of evidence-based treatments (20, 21), outpatient psychotherapy practices in urban and suburban settings are an integral component of the resource pool available to patients with mental health needs. MAACC ultimately developed a regional network of psychotherapy providers to facilitate the navigation of outpatient psychotherapy care with timely psychotherapy referrals and to support the collaborative care model, which has historically focused on building the treatment capacity of primary care providers.

The primary aim of this manuscript was to extend the findings of the MAACC and document support for the program over a 4 years implementation period specific to patient service utilization and short-term treatment outcomes. The secondary aim was to describe how the MAACC developed a regional network of engaged PPCCs and community psychotherapists, to create a patient-centered system of care.

## 2 Method

### 2.1 Participants

#### 2.1.1 Children and adolescents

Patients were referred to MAACC for a mental health evaluation by an MAACC-enrolled pediatrician. PPCCs used screening tools to identify potential diagnostic questions to be evaluated. MAACC Behavioral Health Care Managers (BHCMs) ensured the child met the inclusion criteria: (a) age 6–18 years, (b) primary mental health concern, (c) not previously diagnosed with autism or developmental disorders, and (d) not recently engaged in inpatient or partial hospitalization care that might require more intensive of psychiatric services.

From 2018 to 2022, 1,152 MAACC evaluation referrals were received from MAACC-enrolled PPCCs. Of the patients screened, 696 children and adolescents aged 6–18 (M = 12.12; SD = 3.10) were evaluated and provided treatment recommendations. All patients engaged in MAACC were tracked on a registry to monitor and report treatment progress to associated providers. Of the children and adolescents that participated, 66.8% identified as white, 17.7% of the sample identified as Hispanic, 6.3% as African American, 4.6% as Asian, 3% as biracial, 0.5% as American Indian/Alaskan native, 13.2% as other, and 5.6% declined. Table 1 includes patient demographics.

**Table 1:** MAACC patient characteristics.

Characteristic (*N* = 696)	M (SD)	*n* (%)
Age (years)	12.12 (3.1)	
Gender		
Female		380 (54.6%)
Male		296 (41.9%)
Other		20 (2.8%)
Race		
White		466 (66.8%)
African American		44 (6.3%)
Asian		32 (4.6%)
Biracial		21 (3.0%)
American Indian/Alaskan native		4 (0.6%)
Other		92 (13.2%)
Declined/unknown		39 (5.6%)
Hispanic		122 (17.7%)
Insurance type (Medicaid)*		125 (18%)

Note: *Primary insurance identified as Medicaid in medical record. Secondary Medicaid or public insurance was excluded.

#### 2.1.2 Intervention teams

##### 2.1.2.1 MAACC

The MAACC team includes a BHCM, who screens referrals, schedules evaluations, and monitors patient care through the registry. Child and adolescent psychologists conduct an initial diagnostic evaluation and coordinate with the child and adolescent psychiatrist to provide treatment recommendations. The treatment plan is shared with the patient/parent, PPCC, and therapist. If psychopharmacologic treatment is recommended by the child psychiatrist, the PPCC initiates medication treatment. If therapy recommendations are needed, a list of available providers who can provide appropriate, expedient treatment is provided to the family. The BHCM reviews the registry and progress monitoring data collected from patients with the child psychologist and psychiatrist. This BHCM-guided registry review results in feedback (letters with a review of progress monitoring data) provided to the patient/caregiver, PPCC, and therapist. The child psychiatrist also provides medication consultation as requested by the PPCC. The child psychologist provides diagnostic and psychosocial consultation to the PPCCs and therapists as requested.

##### 2.1.2.2 Pediatric primary care clinicians and practices

The MAACC team approached 145 providers in 39 practices between 2018 and 2022. Three cohorts of practices were engaged initially in June 2018 (Cohort 1), September 2019 (Cohort 2), and May 2020 (Cohort 3). Of the 145 approached providers, including MD, DO, and Advanced Practice Providers, 97 were considered fully trained in the MAACC model. The average number of MAACC referrals by enrolled provider was 8 (SD 10.5) and 23.5 (SD 28.6) by practice. PPCC and practice characteristics are included in Table 2.

**Table 2:** MAACC primary care provider characteristics.

Cohort		1	2	3
Cohort start date		06/2018	09/2019	05/2020
Number of providers		53	27	65
Number of practices		15	5	19
Average practice size (providers)		5.9	8	7.1
Degree	MD	51 (96%)	23 (85%)	57 (88%)
	DO	2 (4%)	2 (7.5%)	6 (9%)
	APP		2 (7.5%)	2 (3%)
Training	Approached	53	27	65
	Enrolled^a^	49 (92%)	19 (70%)	29 (45%)
Location	Urban	2 (4%)	26 (96%)	5 (8%)
	Suburban	51 (96%)	1 (4%)	60 (92%)

Note: APP, advance practice provider.

a Pediatric clinicians are considered enrolled in MAACC if they (a) completed at least 50% of the educational modules, (b) attended a monthly case conference, and (c) referred a patient for MAACC clinical care.

##### 2.1.2.3 Community therapists

To support the psychosocial treatment needs of patients referred to MAACC, a referral network of child and adolescent-focused community therapists was created. The network included 213 licensed providers across a 5-county metropolitan area. The majority of these psychotherapy providers identified prior training in cognitive behavioral therapy (74.6%) or parent management therapy (39.6%). Characteristics of this group are provided in Table 3.

**Table 3:** Outpatient therapy provider characteristics.

Characteristic		*n* (% Sample)	Average (SD)
Degree/certification		213	–
Phd/PsyD		98 (46%)	–
LCSW/APSW		49 (23%)	–
LCP/LCPC/NCC		61 (29%)	–
Other^a^		2 (1%)	–
Identified training in CBT		150 (74.6%)	–
Identified training in PMT		50 (39.6%)	–
Accepted commercial insurance outright		146 (69.1%)	–
Average years in practice			9.98 (8.46)

Note: PhD/PsyD, doctoral degree in psychology; LCSW, licensed clinical social worker, APSW, advanced practice social worker; LPC, licensed professional counselor; LCPC, licensed clinical professional counselor; NCC, national certified counselor; CBT, cognitive behavioral therapy; PMT, parent management therapy.

a Certified alcohol and drug counselor and licensed marriage and family therapist.

### 2.2 Measures

#### 2.2.1 Anxiety disorders interview schedule for DSM-IV: child and parent versions

The ADIS-IV C/P is a semi-structured diagnostic interview that was used to assess psychopathology and confirm diagnostic impressions among children and adolescents aged 6–18 years. There is strong evidence supporting the reliability, validity, and sensitivity to clinical change for the ADIS-IV-C/P (22). The ADIS-IV-C/P modules for attention deficit hyperactivity disorder, oppositional defiant disorder, separation anxiety, generalized anxiety, social anxiety, panic disorder, and depression were administered to all children and adolescents and parents, in addition to measures completed at baseline.

#### 2.2.2 PROMIS anxiety and depression short forms 2.0

The PROMIS measures were used to assess symptoms of anxiety and depression in children (ages 8–17) and parent proxy (ages 5–17) (23, 24). Likert response total scores range from 8 to 40 (1 = “never” to 5 = “almost always”). Summed raw scores and associated T-scores (*M* = 50, SD = 10) are provided on the Health Measures website.^1^ There are no established clinical cutoff scores for the PROMIS A-SF or PROMIS D-SF, although T-score severity levels of mild–moderate and moderate–severe have been described in a large sample (25).

#### 2.2.3 NICHQ Vanderbilt parent rating scale

The NICHQ Vanderbilt (26) is a parent report for children aged 6–12, which was used to measure ADHD (18 items) and ODD (8 items) symptoms. The NICHQ also measures conduct disorder (14 items) and anxiety/depression (7 items) symptoms and includes a school performance and social functioning subscale (8 items). Symptom items are rated using a 4-point Likert scale (never to very often), and the performance items are rated on a 5-point Likert scale from problematic to above average. The NICHQ has favorable psychometrics and is used widely in pediatric primary care (27).

### 2.3 Procedures

#### 2.3.1 Engaging pediatric clinicians and practices in MAACC

Pediatric clinicians were invited to participate in MAACC because of either their involvement in a clinically integrated network or an expressed interest in MAACC directly to program staff. An MAACC psychologist (JP), psychiatrist (CR), and BHCM met with PPCCs and clinic managers in each practice for an introductory meeting to describe the program, referral/consultation process, and expected education engagement if enrolled in MAACC. While all PPCCs in an approached practice were invited to participate, not all providers participated. Our team considered a provider enrolled in MAACC if they (a) completed at least 50% of the educational modules, (b) attended a monthly case conference, and (c) referred a patient for MAACC clinical care. Three distinct waves (Cohort 1, Cohort 2, and Cohort 3) of recruitment took place from June 2018 to May 2022, to monitor the volume of referred patients and available hub staffing.

#### 2.3.2 Engaging community therapists

Community therapists were approached to participate in the MAACC referral system between 2018 and 2022. The goal was to create a referral base of therapists equipped to provide high-quality, timely, and proximate therapy referrals for children and adolescents referred to MAACC. The initial group of therapists was identified by MAACC PPCCs and the Department of Psychiatry internal resource list. Two clinical psychologists (JP or EP) reviewed an initial pool of mental health psychologists and therapists. Therapists primarily treating adults or not engaging in evidence-based treatments for childhood anxiety, depression, or ADHD were excluded from further consideration. From this initial pool, clinicians were contacted for a phone conversation to review and establish training experience (i.e., degree program, child-specific training, and years in practice), typical practice (i.e., population and treatments used), and accessibility (i.e., waitlists and insurance type accepted). Community child and adolescent psychotherapists who reported evidence-based treatments were then invited to complete the survey and join the listserv. The psychotherapy listserv currently functions in 5 regions with 213 licensed providers.

As new therapy referrals were needed, the MAACC clinical team drafted a referral request that was emailed by the BHCM. Non-identifiable information about the patient’s age, diagnosis, prior treatment history, and treatment needs was sent with instructions for therapists to reply if they had intake appointment availability within 3 weeks. Typically, two to three available community psychotherapists were provided to families.

#### 2.3.3 Procedures for evaluation and treatment monitoring

After referral and screening, all patients and parents received the PROMIS A-SF, PROMIS D-SF, and Parent NICHQ Vanderbilt by mail or through a secure portal prior to evaluation. A licensed psychologist administered the ADIS-IV-C/P anxiety modules with the child or adolescent and at least one parent/guardian [see procedures in Parkhurst et al. (18)]. Following evaluation and treatment recommendations, MAACC-enrolled patients were monitored on a registry and engaged in measurement-based care, targeted for their primary diagnosis (i.e., if ADHD was identified, parents received the parent report NICHQ Vanderbilt every 6 weeks from the baseline evaluation). Scheduled measure completion was attempted at 6 weeks intervals for all patients over the course of a year. BHCMs contacted families to prompt measurement-based care measure completion every 8 weeks and assess additional care needs.

Progress monitoring data were analyzed using paired-sample *t*-tests in SPSS (28). We identified mean change and effect sizes from baseline to progress monitoring time points. While progress monitoring was attempted every 6 weeks, there were significant response lags from patients and parents. We analyzed progress monitoring data for any subsequent completed measure. We also elected to group progress monitoring responses by time point at 6 weeks (4–8 weeks response), 12 weeks (10–14 weeks response), and 18 weeks (16–20 weeks response).

## 3 Results

Study procedures and the collection of retrospective data were approved by the Institutional Review Board at Ann and Robert H. Lurie Children’s Hospital.

### 3.1 Patient characteristics, treatment plans, and plan implementation

Key patient descriptive statistics are reported in Table 4.

**Table 4:** MAACC patient diagnosis.

Variable	*n* (%)
Primary diagnosis (*n* = 678)	
ADHD	208 (30.7)
Mood disorder	115 (17.0)
Anxiety disorder	308 (45.4)
Trauma related disorder	23 (3.4)
Disruptive disorder	14 (2.0)
Other	10 (1.4)
Any ADHD diagnosis^a^	284 (41.7)
Any anxiety disorder diagnosis^b^	497 (73.0)
Any mood disorder diagnosis^c^	234 (34.4)
Treatment history	
Prior medication	238 (33.9)
Prior psychotherapy	451 (63.9)
Psychotherapy after enrollment	
New psychotherapy recommended^d^	524 (75.5)
New psychotherapy confirmed	323 (45.6)
Maintained previous psychotherapist confirmed	57 (8.1)
Switched to new psychotherapist confirmed	40 (5.6)
Medication recommendation	
New medication trial	248 (36.9)
Psychostimulant recommendation	126 (51.6)
SSRI recommendation	115 (47.1)
Other recommendation^e^	3 (1.2)
No medication recommendation	265 (39.4)
Medication class change	17 (2.5)
Medication titration	79 (11.8)
No change of current medication(s)	63 (9.4)

Note:

a Any attention deficit disorder diagnosis (including 1^o^, 2^o^, or 3^o^ diagnosis).

b Any anxiety disorder diagnosis including generalized anxiety disorder, panic disorder, social anxiety disorder, separation anxiety disorder, anxiety (NOS; including 1^o^, 2^o^, or 3^o^ diagnosis).

c Any mood disorder diagnosis including major depressive disorder, depression (NOS), and mood disorder (i including 1^o^, 2^o^, or 3^o^ diagnosis).

d Includes those who did not enter MAACC program with a current therapist and recieved a recommendation for therapy, and those who entered with a therapist and were confirmed to be connected to a new therapist.

e Includes alpha agonists, Serotonin–norepinephrine reuptake inhibitor, Norepinephrine reuptake inhibitors, and > 1 class prescribed.

#### 3.1.1 Primary diagnoses

At the evaluation, primary disorders were as follows: anxiety disorder (*n* = 308, 45.4%), ADHD (*n* = 208, 30.7%), mood disorder (*n* = 115, 17.0%), trauma-related disorder (*n* = 23, 3.4%), and disruptive behavior disorder (*n* = 14, 2.0%). One or more comorbid diagnoses were observed in 69.6% (*n* = 472) of patients.

#### 3.1.2 Prior mental health treatment

At the time of evaluation, many patients had received some mental health therapy or medication treatment; 451 children and adolescents had received prior psychotherapy in their history, with 200 (28.9% of the total sample) actively engaged in psychotherapy at the time of evaluation. There were 33.9% (*n* = 238) of patients referred who had previously been treated with a psychotropic medication; of that group, 27.0% (*n* = 187) were actively taking psychotropic medication at the time of evaluation. The most common medications previously trialed were psychostimulants (45.7%, *n* = 86) and selective serotonin reuptake inhibitors (39.9%, *n* = 75).

#### 3.1.3 Medication recommendations

Following the MAACC evaluation, no immediate recommendation for a psychotropic medication was made for 39.4% (*n* = 265) of patients. A new medication trial (for children who had not been medicated previously for a mental health problem) was recommended for 36.9% (*n* = 248) of patients; an increased dose of the child’s current medication for 11.8% (*n* = 79) of patients; a medication class change for 2.5% (*n* = 17) of patients; and a continuation of current medication with no change for 9.4% (*n* = 63) of patients.

#### 3.1.4 Psychotherapy recommendations

From evaluation, new therapy recommendations were provided for 75.5% (*n* = 524) of evaluated patients. The BHCM was able to confirm that 45.6% (*n* = 323) of patients connected with new therapists and that 8.1% (*n* = 57) remained engaged with a psychotherapist that the patient was working with prior to evaluation. For 19.2% (*n* = 136) of cases, coordinators were unable to confirm the connection with a therapist despite the patient and parent receiving referrals.

### 3.2 Patient outcome measures

Progress monitoring data were collected for children and adolescents during the initial 18 weeks post-evaluation, with measures provided at weeks 6, 12, and 18. In total, 401 (58.2%) children and adolescents completed the first progress monitoring measure, 254 (38%) children and adolescents completed the second progress measure, and 186 (28.8%) children and adolescents completed the third measure. The median time between evaluation and the completion of at least one follow-up measure was 13 weeks for the PROMIS and 17 weeks for the Vanderbilt.

#### 3.2.1 Anxiety

Of the 465 children and adolescents with anxiety, 247 parents and 211 children and adolescents completed at least one PROMIS A-SF measure post-baseline (referred to herein as “first follow-up”). Parents (*n =* 247, *d* = 0.14, *p* = 0.018) and children and adolescents (*n* = 211, *d* = 0.2, *p* = 0.002) noted a significant difference between baseline and first follow-up (Table 5). Overall, the effect size for improvement to the first follow-up measure was small. For children and adolescents with any anxiety who returned a PROMIS A-SF measure at a specific follow-up window (6, 12, or 18 weeks) (Table 6), significant improvement with small to moderate effect size was observed from baseline to 6 weeks on parent (*n* = 60, *d =* 0.29, *p* = 0.014) and children and adolescents (*n* = 61, *d* = 0.42, *p* = <0.001) measures; baseline to 12 weeks on parent (*n* = 74, *d* = 0.41, *p* = <0.001) and children and adolescents (*n* = 68, *d* = 0.50, *p* = <0.001) measures; and baseline to 18 weeks on parent (*n* = 51, *d* = 0.34, *p* = 0.010) and children and adolescents (*n* = 47, *d* = 0.47, *p* = 0.001) measures.

**Table 5:** MAACC population measurement-based care change.

Outcome	Baseline		First follow-up		
	*N*	*M*	*M*	*d*	*p*
Vanderbilt					
ADHD-I^a^	156	17.6 (0.54)	15.99 (0.46)	0.29 [0.130, 0.451]	<0.001***
ADHD-H^b^	156	11.70 (0.55)	10.65 (0.53)	0.21 [0.047, 0.364]	0.006**
ADHD-C^c^	156	29.30 (0.83)	26.65 (0.84)	0.28 [0.118, 0.438]	<0.001***
PROMIS					
Depression					
Parent	115	17.46 (0.526)	15.65 (0.526)	0.33 [0.141, 0.516]	<0.001***
Youth	108	28.19 (0.768)	25.03 (0.839)	0.33 [0.132, 0.520]	<0.001***
Anxiety					
Parent	247	19.91 (0.454)	18.97 (0.402)	0.14 [0.009, 0.260]	0.018*
Youth	211	22.68 (0.539)	21.12 (0.519)	0.20 [0.061, 0.334]	0.002**

Standard errors are in parentheses. 95% confidence intervals are in brackets. A positive mean difference value indicates an improvement in symptoms. The sample size decreases over time from baseline to 18 weeks as noted by the change in *n* due to attrition. The Vanderbilt was parent-reported. For the Vanderbilt ADHD measure *t*-test, the sample only includes patients who were diagnosed with any ADHD diagnosis (including a 1^o^, 2^o^, 3^o^, or 4^o^ diagnosis). For the PROMIS Depression measure *t*-test, the sample only includes patients who were diagnosed with any mood disorder (including a 1^o^, 2^o^, 3^o^, or 4^o^ diagnosis). For the PROMIS Anxiety measure, the sample only includes patients who were diagnosed with any anxiety disorder (including a 1^o^, 2^o^, 3^o^, or 4^o^ diagnosis). * *p* < 0.05; ** *p* < 0.01; *** *p* < 0.001.

a ADHD inattentive.

b ADHD hyperactive.

c ADHD combined.

**Table 6:** MAACC population measurement-based care change.

Outcome	Baseline		6 weeks				Baseline	12 weeks				Baseline	18 weeks		
	*N*	*M*	*M*	M*diff*	*p*	*N*	*M*	*M*	M*diff*	*p*	*N*	*M*	*M*	M*diff*	*p*
Vanderbilt															
ADHD-I^a^	28	17.21 (1.0)	15.54 (0.9)	1.679 (0.98)	0.049*	34	17.59 (0.92)	16.62 (0.91)	0.971 (0.64)	0.069	34	16.24 (1.11)	13.94 (1.01)	2.294 (1.19)	0.031*
ADHD-H^b^	28	11.71 (1.39)	11.54 (1.35)	0.179 (1.02)	0.451	34	10.97 (1.02)	9.24 (0.95)	1.735 (0.68)	0.008**	34	12.12 (1.12)	10.71 (1.03)	1.412 (1.15)	0.115
ADHD-C^c^	28	28.93 (2.09)	27.07 (1.80)	1.857 (1.74)	0.147	34	28.56 (1.54)	25.85 (1.47)	2.706 (1.10)	0.010*	34	28.35 (1.81)	24.65 (1.74)	3.706 (2.19)	0.050
PROMIS															
Depression															
Parent	33	16.00 (1.00)	12.76 (0.934)	3.242 (1.00)	0.001**	34	17.91 (1.02)	14.00 (1.16)	3.912 (1.06)	<0.001***	28	17.89 (1.06)	14.04 (1.28)	3.857 (1.28)	0.003**
Youth	31	27.42 (1.30)	22.81 (1.92)	4.613 (1.96)	0.013*	33	29.00 (1.40)	22.76 (1.95)	6.242 (1.94)	0.001**	26	28.27 (1.88)	20.12 (2.05)	8.154 (2.68)	0.003**
Anxiety															
Parent	60	19.82 (0.89)	17.65 (0.85)	2.167 (0.97)	0.014*	74	19.89 (0.87)	17.08 (0.83)	2.811 (0.79)	<0.001***	51	19.73 (1.10)	16.57 (1.11)	3.157 (1.30)	0.010*
Youth	61	22.67 (0.92)	19.28 (1.29)	3.393 (1.03)	<0.001***	68	22.74 (0.90)	18.24 (1.06)	4.500 (1.09)	<0.001***	47	21.85 (0.97)	16.91 (1.47)	4.936 (1.54)	0.001**

Standard errors are in parentheses. A positive mean difference value indicates an improvement in symptoms. The sample size decreases over time from baseline to 18 weeks as noted by the change in *n* due to attrition. The Vanderbilt was parent-reported. For the Vanderbilt ADHD measure *t*-test, the sample only includes patients who were diagnosed with any ADHD diagnosis (including a 1^o^, 2^o^, 3^o^, or 4^o^ diagnosis). For the PROMIS Depression measure *t*-test, the sample only includes patients who were diagnosed with any mood disorder (including a 1^o^, 2^o^, 3^o^, or 4^o^ diagnosis). For the PROMIS Anxiety measure, the sample only includes patients who were diagnosed with any anxiety disorder (including a 1^o^, 2^o^, 3^o^, or 4^o^ diagnosis). * *p* < 0.05; ** *p* < 0.01; *** *p* < 0.001.

a ADHD inattentive.

b ADHD hyperactive.

c ADHD combined.

#### 3.2.2 Depression

For children and adolescents diagnosed with a mood disorder (*n* = 218), parents (*n* = 115, *d* = 0.33, *p* = 0.018) and children and adolescents (*n* = 108, d = 0.33, *p* = 0.002) noted a significant difference between baseline to the first follow-up PROMIS D-SF. Overall, the effect sizes were small to medium in magnitude but larger for depression than anxiety. Medium to large effects were observed for children and adolescents with mood disorders from baseline to 6 weeks on parent (*n* = 33, *d* = 0.57, *p* = 0.001) and children and adolescents (*n* = 31, *d =* 0.43, *p* = 0.013) measures; baseline to 12 weeks on parent (*n* = 34, *d* = 0.63, *p* = <0.001) and children and adolescents (*n* = 33, *d* = 0.56, *p* = 0.001) measures; and baseline to 18 weeks on parent (*n* = 28, *d* = 0.57, *p* = 0.003) and children and adolescents (*n* = 26, *n* = 0.60, *p* = 0.003) measures.

#### 3.2.3 ADHD

For the 273 children and adolescents with ADHD, there were 156 subsequent parent NICHQ Vanderbilts that were completed. Significant improvements with small effect sizes were noted for parent-reported inattention (*n* = 156, *d* = 0.29, *p* = 0.001), hyperactivity (*n* = 156, *d* = 0.21, *p* = 0.006), and combined (*n* = 156, *d* = 0.28, *p* = 0.001). For children and adolescents with multiple follow-up measures, significant improvement was observed from baseline to 6 weeks on inattentive symptoms (*n* = 28, *d* = 0.32, *p* = 0.049); baseline to 12 weeks on hyperactive (*n* = 34, *d* = 0.44, *p* = 0.008) and combined (*n* = 34, *d* = 0.42, *p* = 0.010) measures; and baseline to 18 weeks on inattentive (*n* = 34, *d* = 0.33, *p* = 0.031) measures.

#### 3.2.4 Psychotherapy only versus combined medication/psychotherapy

Outcomes were assessed for children and adolescents with any anxiety. Those who were both confirmed to have been connected to a therapist and confirmed to have been prescribed medication had a greater change in their children and adolescents anxiety measures than those who were only confirmed to have been connected to a therapist [*t*(99) = 1.911, *p* = 0.029, one-tailed]. There were no observed differences for children and adolescents with depression or ADHD in comparison between patients receiving therapy alone or therapy and medication.

## 4 Discussion

MAACC is a pediatric hospital-based collaborative care program, designed to build mental health capacity for small- to medium-sized pediatric primary care practices. The hub-and-spoke MAACC model connected children from multiple local primary care practices to evidence-based evaluations, delivered medication management recommendations to the child’s PPCC, and linked the child and family to psychotherapy care. Patient-level outcomes indicate that effective treatment of child and adolescent depression, anxiety, and ADHD can be achieved through pediatric practices working with a hospital-based collaborative program connected to a network of community therapy providers.

MAACC was developed to support pediatric primary care practices that were small (average practice size of seven providers) and managed independently from the hospital system. PPCC participation in MAACC was a choice: Of the 145 pediatric clinicians approached across 39 practices, 97 (66%) enrolled in MAACC. The average number of referrals to MAACC by enrolled providers was 8. Cohort 3 had the largest number of PPCCs that did not enroll in MAACC. Several factors may have impacted the choice of PPCCs to engage in MAACC, including the perceived value of MAACC participation, proximity of the MAACC treatment hub to individual practice, and challenges to practice engagement engendered by the COVID pandemic (especially for Cohort 3).

A large group of child and adolescent psychotherapists were identified and participated in a referral listserv. This listserv was used to provide referrals to a new psychotherapist for 75.5% (*n* = 524) of evaluated patients. Through the creation of this regional network of psychotherapy providers, we had immediate knowledge of psychotherapy provider availability, insurance acceptability, and proximity of provider’s practice to patient’s home, thereby increasing the likelihood of patient receipt of expedient evidence-based psychotherapy.

MAACC patient-level outcomes extended prior data with a much larger sample. Referrals to our team included children and adolescents with anxiety, depression, and/or ADHD symptoms. Adolescents with internalizing concerns were the most common referral. There was a high comorbidity in our sample, which reflects similar community samples. Many (63.9%) of the referred patients had prior experience in therapy and (33.9%) with prior psychoactive medication. Following evaluation and diagnosis, our clinical team of a psychologist and psychiatrist provided new psychotherapy recommendations (75.5%) and new medication recommendations (36.6%) for the children and adolescents who had been evaluated.

Progress monitoring data were collected specifically for the diagnosis. Despite program attrition, most patients and caregivers completed at least one additional progress monitoring measure. There were significant improvements in parent- and children and adolescents-reported symptoms of anxiety from baseline evaluation to 6, 12, and 18 weeks. The largest effect size was observed in parent- and children and adolescents-reported outcomes for children and adolescents with mood disorders at 6, 12, and 18 weeks. Parent-reported ADHD-inattentive symptoms improved from baseline to 6 and 18 weeks. Patients who received medication and therapy displayed significant improvement over those who received therapy alone for children and adolescents with anxiety disorders.

### 4.1 Limitations

Improved access to mental health treatment will require the engagement of the larger pediatric primary care clinician workforce. In MAACC, we observed that 34% of approached providers chose not to engage in the program, with a large proportion of the unengaged providers coming from Cohort 3. Understanding reasons for or barriers to engagement in MAACC may lead to a better understanding of the phenotype of PPCCs who uptake collaborative care models. Furthermore, while collaborative care models may drive access to mental healthcare in primary care, there are unexamined questions that remain related to the quality of this care. For example, we identified that if therapy and medication were initiated and patient progress was monitored, the anxiety and mood disorders of children and adolescents improved; however, we have not investigated whether PPCC prescribing met practice parameters or if medication dose titration was tied to progress monitoring outcomes effectively, despite active consultation from the MAACC team. Similarly, while our team cultivated a network of psychotherapy providers, we did not systematically monitor psychotherapy treatment objectives and evidence-based care implementation.

The MAACC collaborative care model demonstrated symptom improvement through progress monitoring data and follow-up care coordination. Patient attrition in the program and collection of progress monitoring tools over time was challenging. Despite our efforts to make progress monitoring measures accessible (mail, web, and health record portal) and follow-up and feedback provided to families and PPCPs proactive, families were inconsistent in the completion of progress monitoring measures. A large group of children and adolescents and families were delayed in timely response to measures or opted out of progress monitoring altogether. Furthermore, there are some families that are referred from their PPCCs to MAACC, but do not follow through with completing the initial evaluation or are not appropriate for MAACC programming (e.g., already connected to appropriate services and require a more intensive level of services). There is a need to address potential barriers to accessing MAACC services for families and to support PPCCs in identifying appropriate referrals.

MAACC may not generalize to other regions or populations. The MAACC model was developed with the acknowledgment of local resources that may not be available outside of urban or suburban regions. Additionally, our patient sample reflected the demographic and socioeconomic features of the practices we engaged in MAACC. Only 18% of our sample has primary Medicaid insurance. Thus, the use of a regional collaborative care model, such as MAACC, may only effectively work when a large number of referred patients have fewer barriers to psychotherapy treatment (e.g., commercial insurance and proximity to community psychotherapy providers).

### 4.2 Clinical implications and future directions

The core components of collaborative care that have been linked to the best clinical outcomes are population-based care, measurement-based care, and access to evidence-based mental health services (14). The implementation and delivery of components require further investigation. While MAACC demonstrated patient symptom improvement, there were clearly real-world challenges that impacted the effectiveness of treatment, including completeness of measurement-based care and being able to verify evidence-based psychotherapeutic and medication treatment. Program attrition was also high. Patient improvement coupled with low engagement may suggest that “lighter touch” collaborative models may still achieve patient-centered goals while reducing costs. Identifying children and adolescents and parent perceptions of valued aspects of collaborative care and patient factors may result in optimal benefits with minimal programming. For example, children and adolescents and parents who have been directed to appropriate services may not need frequent, proactive contact from BHCMs. Furthermore, it is apparent that some children and adolescents and parents find measurement-based care metrics too onerous, frequent, or without direct benefit to them. Research is needed to understand how to increase the completeness of measurement-based care and the optimal timing of measurement-based care to support clinical utility while reducing patient burden.

Collaborative models were developed to provide access to high-quality clinical services in primary care. Collaborative models also advance the capacity of primary care physicians through training and consultation. An understanding of how physician engagement in collaborative care programming shapes future treatment is needed, as it is likely that PPCCs trained in collaborative care models continue providing mental healthcare after they have stopped referring or engaging in collaborative care models. Collaborative care models, such as MAACC, may also have an opportunity to assess the quality of evidence-based medication treatment provided. Researchers have found strategies to improve psychotropic medication management in primary care (29), but further investigation is needed for collaborative models to enhance evidence-based medication treatment.

Similarly, the creation and facilitation of regionally available therapy resources by a collaborative care program is novel. Confirming community provider use of evidence-based practices is a challenge without established base-level training and consultative support structure. However, the components of collaborative care inherently align well with the objectives of many outpatient therapy providers. Enhanced communication, training, and consultation between community psychotherapists and the MAACC may improve a high-quality regional network of mental healthcare.

Collaborative care billing codes are becoming more commonly used but remain challenging to implement for many pediatric practices due to the heavy administrative burden of tracking time in coordination. The generally low Medicaid reimbursement rates also impact the feasibility of effective reimbursement to offset services.

## 5 Conclusion

MAACC adapts to primary care practice needs and can support patient outcomes through regionally available resources. This collaborative care iteration has had the unanticipated effect of building increased structure and communication among often siloed professionals in the community. In positioning the program between pediatric clinician cohorts and a network of psychotherapists providing child-focused, evidence-based care, MAACC optimizes the mental health treatment for children and adolescents seeking care in their pediatric medical home.

## Data availability statement

The raw data supporting the conclusions of this article will be made available by the authors, without undue reservation.

## Ethics statement

The studies involving humans were approved by Ann and Robert H. Lurie Children’s Hospital. The studies were conducted in accordance with the local legislation and institutional requirements. The ethics committee/institutional review board waived the requirement of written informed consent for participation from the participants or the participants’ legal guardians/next of kin because the IRB identified that de-identified data could be reported retrospectively without harm to patient confidentiality.

## Author contributions

JP conceived, designed, analyzed data, and wrote the draft of this manuscript. CG-G analyzed data and drafted the manuscript. EP collected data and drafted the manuscript. CR collected data and reviewed the manuscript. RB and JL reviewed and provided thorough edits for this manuscript. All authors contributed to the article and approved the submitted version.

## Footnotes

^1^https://www.healthmeasures.net/search-view-measures?task=Search.search
